# Three Signs of a Shortcut: Gastrogastric Fistula Permitting Simple Roux-en-Y Endoscopic Retrograde Cholangiopancreatography

**DOI:** 10.14309/crj.0000000000000694

**Published:** 2021-11-03

**Authors:** Matthew P Heinrich, Andrew C Storm

**Affiliations:** 1 Department of Internal Medicine, Mayo Clinic School of Graduate Medical Education, Rochester, MN; 2 Division of Gastroenterology and Hepatology, Department of Medicine, Mayo Clinic, Rochester, MN

## CASE REPORT

**Figure 1.**
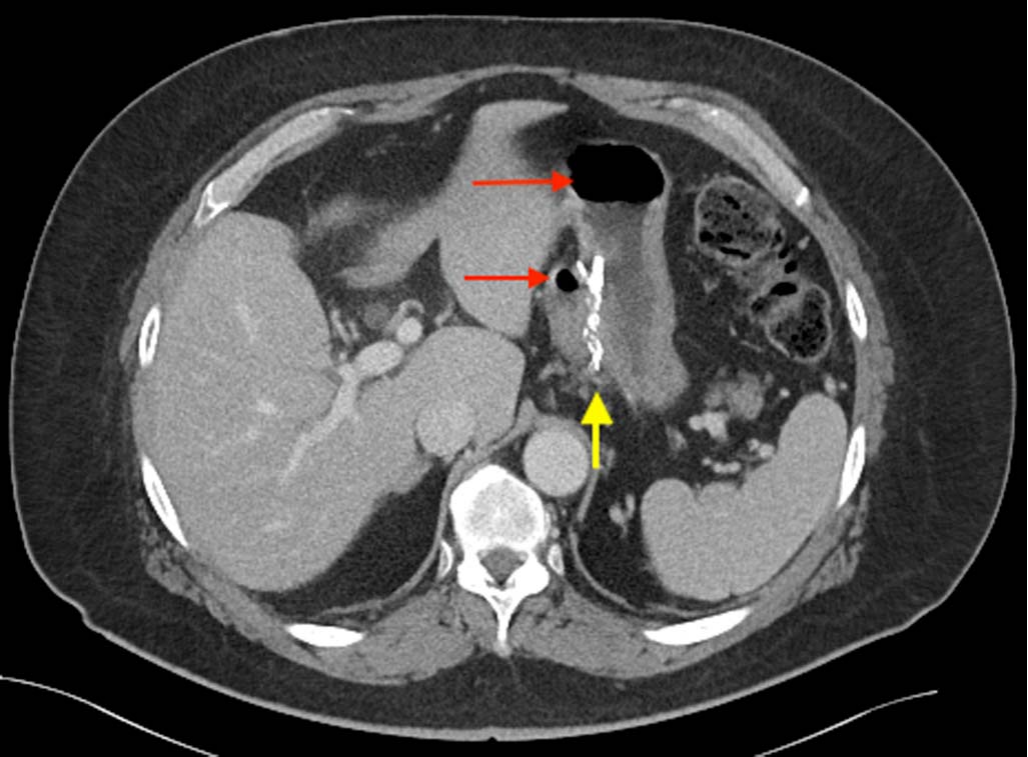
The staple line (yellow arrow), which separates the gastric pouch to the left and remnant stomach to the right, suggests a nondivided surgical technique was used at the time of gastric bypass, increasing the risk of fistula formation. Note the presence of air in both the gastric pouch and remnant stomach (red arrows).

**Figure 2.**
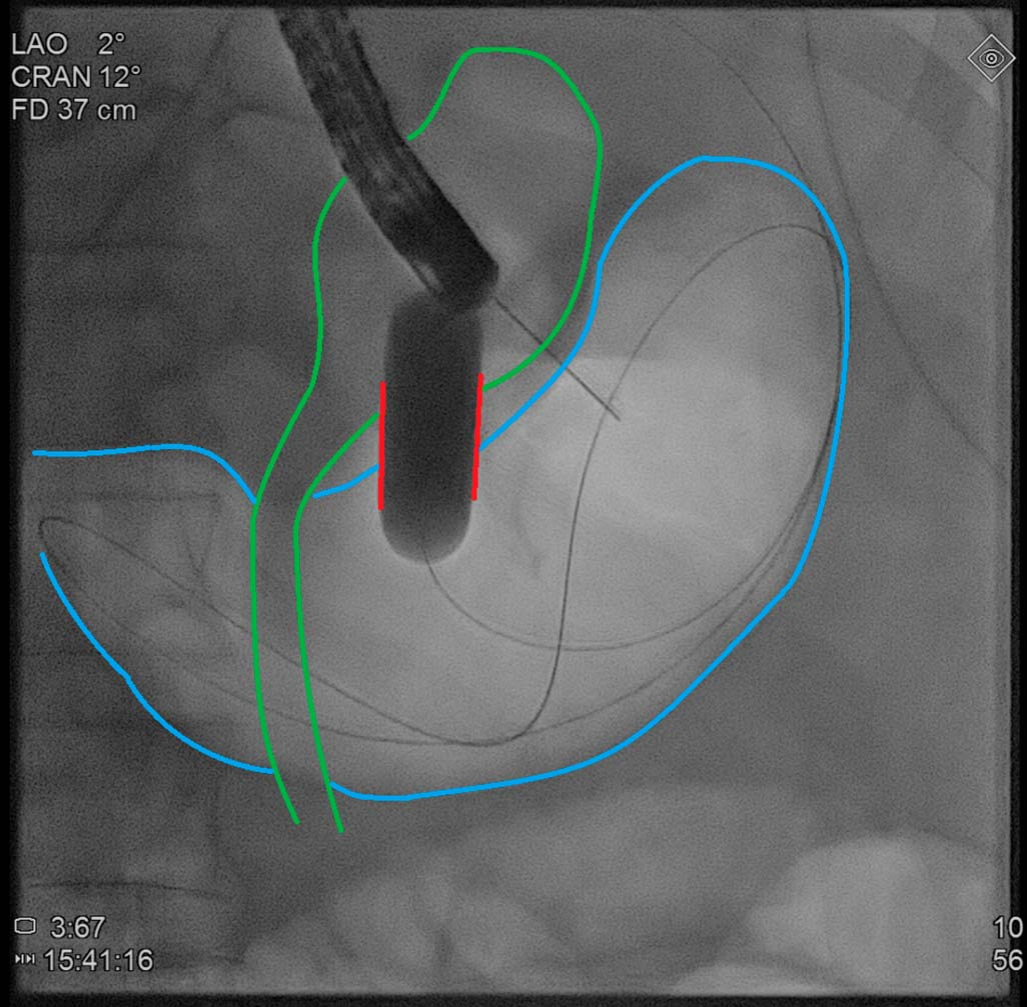
Dilation of the fistula (red lines) connecting gastric pouch (green outline) to remnant stomach (blue outline) with a 12- to 15-mm balloon catheter under fluoroscopic guidance.

**Figure 3.**
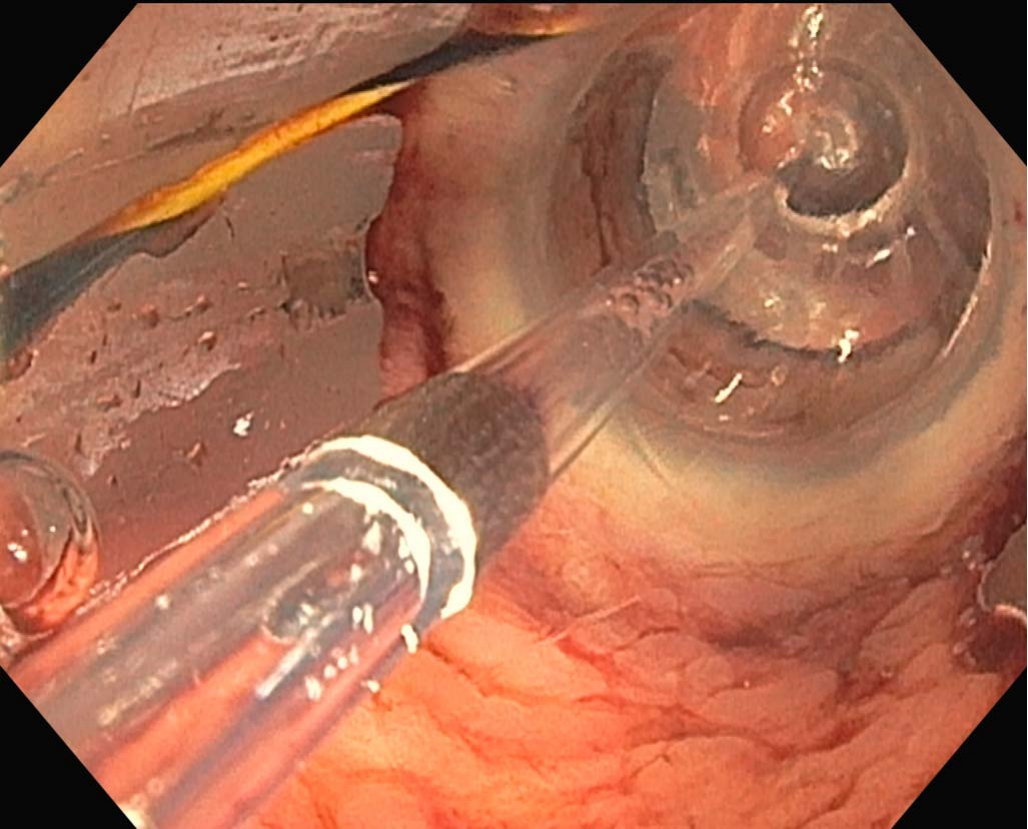
Endoscopic view of the gastrogastric fistula dilated with a 12- to 15-mm balloon catheter.

**Figure 4.**
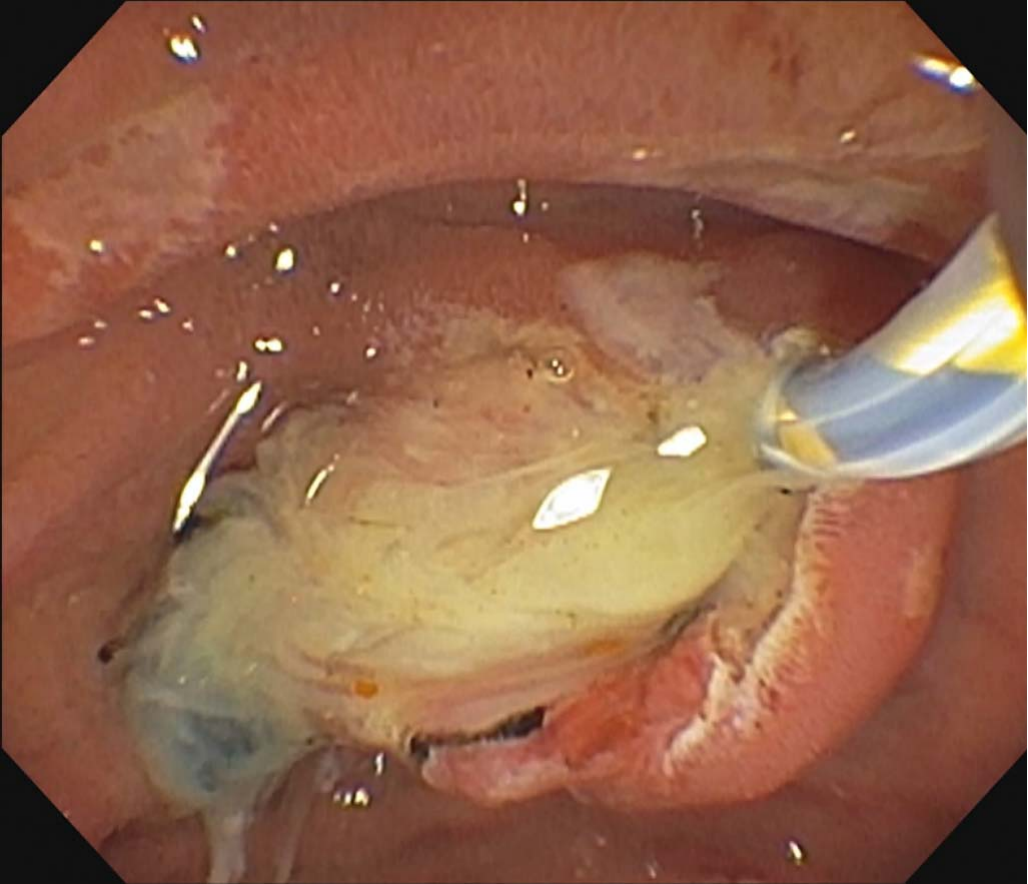
The biliary tree was swept with an 11.5-mm balloon with removal of 3 black pigment stones.

A 68-year-old man with obesity (body mass index of 36) and history of Roux-en-Y gastric bypass with cholecystectomy in 1980 presented with fever, nausea, and right upper-quadrant abdominal pain. Abdominal computed tomography demonstrated stones in the distal common bile duct. Three signs from computed tomography suggest the presence of a gastrogastric fistula including air in the remnant stomach, excess adiposity (suggesting either poor weight loss or weight regain since bypass), and staple line immediately between the gastric pouch and remnant stomach, suggesting a nondivided surgical technique (Figure 1). Endoscopy was then performed, which confirmed presence of a subtle gastrogastric fistula. The fistula was then dilated, allowing for passage of a duodenoscope without the need for device-assisted enteroscopy endoscopic retrograde cholangiopancreatography (ERCP) or endoscopic ultrasound–directed transgastric access as would otherwise be required to complete this ERCP in the setting of surgically altered anatomy (Figure 2 and 3). The duodenoscope was advanced to the second portion of the duodenum to visualize the major papilla. The bile duct was then swept with a 11.5-mm balloon, which extracted several black pigment stones and a moderate amount of pus (Figure 4). On completion of the procedure, the gastrogastric fistula was left in place to allow access should further intervention be needed including treatment of postsphincterotomy bleeding, which did not occur. The patient experienced resolution of his abdominal pain after the procedure and was discharged 2 days later without complication.

Gastrogastric fistula is an uncommon complication of Roux-en-Y gastric bypass that often presents with weight regain and abdominal pain.^1^ Although contrast studies or direct visualization on esophagogastroduodenoscopy is necessary to confirm its presence, findings on standard abdominal computed tomography such as air in the remnant stomach pouch should raise suspicion for gastrogastric fistula.^1^ Although both surgical and endoscopic approaches to ERCP in patients with Roux-en-Y gastric bypass have been developed, they either require significant time and resources or are technically difficult.^2^ For example, Inamdar et al found single balloon enteroscopy–assisted ERCP in these patients to be successful in merely 61.7% of cases.^3^ Thus, identification of gastrogastric fistula by suggestive findings on abdominal imaging in the correct clinical context can provide an opportunity for more straightforward biliary access through anterograde ERCP and may prevent unnecessary tedious interventions such as device-assisted ERCP or percutaneous biliary drainage.

## DISCLOSURES

Author contributions: M. Heinrich wrote the manuscript, reviewed the literature, and approved the manuscript. A. Storm edited the manuscript, provided the images, and is the article guarantor.

Financial disclosure: A. Storm is a consultant for GI Dynamics, EnteraSense, ERBE, Endo-TAGSS, and Apollo Endosurgery. He holds research grants from Endo-TAGSS, Apollo Endosurgery, and Boston Scientific.

Informed consent was obtained for this case report.
